# The Molecular Basis of Polycystic Ovary Syndrome and Its Cardiometabolic Correlates: Exploring the Intersection and Its Clinical Implications—A Narrative Review

**DOI:** 10.3390/biomedicines13030709

**Published:** 2025-03-13

**Authors:** Jasmin Mahabamunuge, Nicole M. Sekula, Christina Lepore, Meghana Kudrimoti, Animesh Upadhyay, Khadija Alshowaikh, Howard J. Li, David B. Seifer, Abdelrahman AlAshqar

**Affiliations:** 1 Department of Obstetrics, Gynecology and Reproductive Sciences, Yale School of Medicine, New Haven, CT 06510, USA; jasmin.mahabamunuge@yale.edu (J.M.); nicole.sekula@yale.edu (N.M.S.); christina.lepore@yale.edu (C.L.); meghana.kudrimoti@yale.edu (M.K.); animesh.upadhyay@yale.edu (A.U.); khadija.alshowaikh@yale.edu (K.A.); 2 Division of Reproductive Endocrinology and Infertility, National Institutes of Health, Bethesda, MD 20892, USA; howard.li@nih.gov; 3 Division of Reproductive Endocrinology and Infertility, Yale School of Medicine, New Haven, CT 06510, USA; david.seifer@yale.edu

**Keywords:** cardiovascular disease, diabetes mellitus, hypertension, metabolic syndrome, obesity, polycystic ovary

## Abstract

Recent studies have highlighted the association between polycystic ovary syndrome (PCOS) and cardiometabolic diseases, leading to an improved understanding of the underlying mechanistic factors. PCOS significantly increases cardiovascular risk by predisposing individuals to various subclinical and clinical conditions, including atherosclerosis and type 2 diabetes mellitus. Additionally, it interacts synergistically with other traditional cardiovascular risk factors, such as obesity, hyperlipidemia, and insulin resistance. Several molecular mechanisms involving genetics, epigenetics, adipokine secretion, hyperandrogenemia, and hyperinsulinemia play a role in the relationship between PCOS and these comorbidities. For instance, androgen excess has been implicated in the development of hypertension, type 2 diabetes mellitus, endothelial dysfunction, and ultimately, broader cardiovascular disease. A deeper understanding of these underlying mechanisms facilitates the development of diagnostic, preventative, and therapeutic strategies directed at reducing cardiometabolic morbidity. This narrative review summarizes the current evidence, explores the potential clinical implications of these findings, and discusses emerging therapies to reduce cardiometabolic morbidity in women with PCOS.

## 1. Introduction

Polycystic ovary syndrome (PCOS) is the most prevalent endocrinopathy among women of reproductive age, with reported prevalence rates ranging from 10% to 20%, depending on the diagnostic criteria used [1,2]. In the United States, the Rotterdam criteria are the most inclusive criteria and are commonly employed, while the more specific NIH criteria are often utilized in Europe [3]. PCOS is a major cause of morbidity, increasing the risk of a range of reproductive, cardiometabolic, obstetric, and psychiatric complications. These comorbidities include menstrual irregularities, infertility, metabolic syndrome, cardiovascular disease (CVD), obstructive sleep apnea, gestational diabetes, pre-eclampsia, and depression [4]. This broad spectrum of clinical manifestations reflects the complex pathophysiology of PCOS, which involves intricate mechanisms leading to insulin resistance, hyperinsulinemia, and hyperandrogenism [5].

PCOS presents heterogeneously across individuals, with different combinations of diagnostic criteria resulting in four distinct phenotypes [6]. Phenotype A is characterized by hyperandrogenism, ovulatory dysfunction, and polycystic ovaries; phenotype B includes hyperandrogenism and ovulatory dysfunction; phenotype C represents ovulatory PCOS, with hyperandrogenism and polycystic ovaries; and phenotype D is non-hyperandrogenic PCOS, with ovulatory dysfunction and polycystic ovaries. The first two phenotypes are considered classical PCOS, while the latter two are classified as nonclassical PCOS [6].

**Figure 1:**
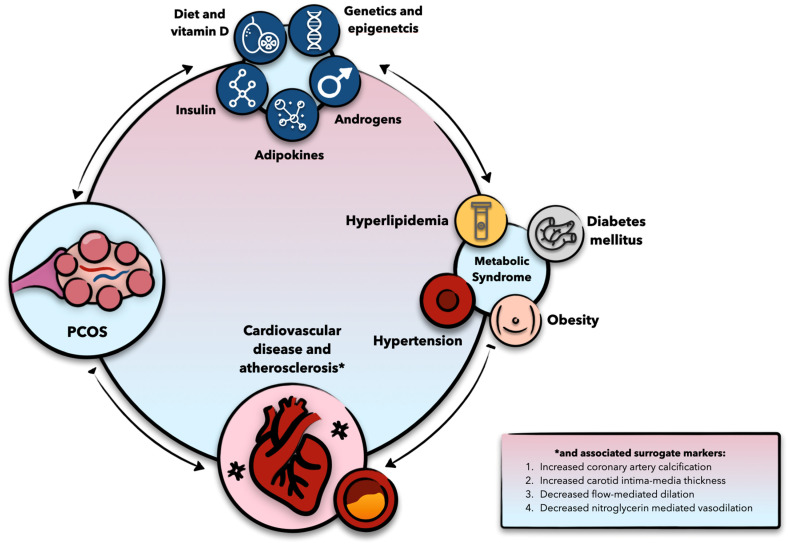
Diagram summarizing the molecular aspects contributing to both polycystic ovary syndrome (PCOS) and cardiovascular risk factors, and in turn to cardiovascular disease, atherosclerosis, and other surrogate markers of both.

Although PCOS is traditionally viewed as a reproductive disorder, it has increasingly been recognized as a cardiovascular risk factor, contributing to accelerated CVD (Figure 1) [3,7]. This risk is present across all PCOS phenotypes but is most pronounced in women with the hyperandrogenic forms (A, B, and C) [8]. This observation underscores the role of androgen excess in elevating cardiovascular risk [9]. However, the etiopathogenesis of both clinical and subclinical CVD in women with PCOS is multifactorial, involving genetic and epigenetic factors, oxidative stress, inflammation, and various environmental influences. Notably, the interaction between androgen excess and hyperinsulinemia plays a particularly synergistic role in amplifying cardiovascular risk, independently of obesity. Despite advances in understanding the pathogenesis of PCOS and its association with cardiometabolic disorders, the precise mechanistic aspects remain poorly understood. This review examines the molecular mechanisms underlying the relationship between PCOS and cardiometabolic diseases and discusses both established and novel diagnostic, preventative, and therapeutic approaches addressing this association.

## 2. Methodology and Search Criteria

We conducted a thorough review of the literature, focusing on the molecular aspects of the intersection between PCOS and CVD. A literature search was conducted using electronic databases, including PubMed of the National Library of Medicine, Embase, Web of Science, and Google Scholar. The keywords “polycystic ovary syndrome”, “insulin”, “androgens”, “sex hormones”, “adipokines”, and “antimüllerian hormone” combined with “cardiovascular disease”, “hypertension”, “diabetes mellitus”, “cardiometabolic”, and “atherosclerosis” were used. The selected studies encompassed a range of research designs from retrospective analyses to prospective observational studies and meta-analyses, and spanned data up to the year 2024. Each study was evaluated for its contribution to the understanding of PCOS and cardiometabolic diseases. All relevant reports were retrieved, and the corresponding reference lists were systematically searched to identify any additional studies that could be included and reviewed. Only high-quality and full-length peer-reviewed publications in English were considered.

## 3. PCOS and Cardiometabolic Disease: Epidemiological Insights

As previously discussed, PCOS has long been associated with a range of cardiometabolic disorders, including insulin resistance, hypertension, myocardial infarction, and stroke (Figure 1) [3]. Metabolic syndrome, a collection of cardiometabolic risk factors that indicates an elevated risk of CVD, is also more prevalent in women with PCOS, occurring 4.5 times more than in age-matched women without PCOS [3,10]. Among women with PCOS, 21.7% meet the criteria for metabolic syndrome when classified using the Rotterdam criteria, compared to 26.9% under the NIH criteria [9]. This discrepancy may be due to the more inclusive nature of the Rotterdam criteria, which identifies a broader range of individuals, including those with oligo- or anovulation and polycystic ovaries but without clinical or biochemical hyperandrogenism. Women diagnosed with the Rotterdam criteria are hypothesized to be less prone to significant insulin resistance and its related metabolic consequences compared to those diagnosed with stricter criteria, where hyperandrogenism is considered to be a mandatory component [3].

A systematic review and meta-analysis examining the long-term cardiometabolic risks associated with PCOS revealed a significantly increased risk of hypertension (risk ratio (RR) 1.75, 95% confidence interval (CI) 1.42–2.15) and type 2 diabetes (RR 3.00, 95% CI 2.56–3.51) in women with PCOS [1]. Additionally, these women exhibited higher serum levels of total cholesterol (mean difference (MD) 7.14 mg/dl, 95% CI 1.58–12.70 mg/dl), lower serum concentrations of high-density lipoprotein (HDL) cholesterol (MD −2.45 mg/dl, 95% CI −4.51 to −0.38), and an increased risk of non-fatal cerebrovascular events (RR 1.41, 95% CI 1.02–1.94) compared to women without PCOS [1]. However, no definitive significant increase in coronary disease events was observed despite the association nearing statistical significance (RR 1.78, 95% CI 0.99–3.23).

At the subclinical level, PCOS is associated with a twice-as-high incidence of coronary artery calcification compared to controls, even among lean patients with PCOS [3,11]. Furthermore, in young women with PCOS, increased carotid intima-media thickness correlates with higher androgen levels and lower insulin sensitivity, independently of body mass index (BMI) [3,12]. Population-based studies on the risk of cardiac events in PCOS present mixed findings; however, a meta-analysis suggests a 21.5-fold-increased RR for coronary heart disease and stroke in women with PCOS after adjusting for BMI [3,13]. In prognostic models that account for factors, such as diabetes, waist circumference, hypertension, and coronary artery disease, PCOS remained a significant predictor of adverse cardiovascular outcomes [3,9]. These findings provide a quantifiable risk assessment that is crucial for clinicians in predicting cardiovascular risk in women with PCOS.

## 4. Molecular Considerations

### 4.1. Genomics and Epigenomics

PCOS is highly heritable, highlighting the significant role of genomics in its development. A large study of a national twin registry of 1332 monozygotic and 1873 dizygotic twins estimated the heritability of PCOS to be as high as 70% [14]. This is consistent with a familial clustering study of first-degree relatives of women affected by PCOS, which found a prevalence of 24% among mothers and 32% among sisters of women with PCOS [15]. Despite the strong population-based evidence of a highly genetic component to PCOS pathogenesis, candidate loci identified by genome-wide association (GWA) studies have only identified less than 10% of heritable risk [16]. One major barrier to genetic discovery has been the highly heterogenous nature of the disorder, partially due to its multi-system manifestations. Indeed, while the most recent guidelines on PCOS diagnosis include some component of androgen excess, ovulatory dysfunction, and polycystic ovarian morphology, expert consensus has held that no single feature is essential to achieving this syndromic diagnosis [17].

Of the genetic loci implicated by GWA studies, multiple hits have highly plausible loci connecting PCOS diagnosis to its cardiometabolic connections. A landmark GWA study of 10,074 PCOS cases and 103,164 controls of European ancestry identified 14 independent loci, including variants associated with genes involved in insulin signaling (*INSL4*, *INSL6*) and genes associated with adipocyte function, lipid metabolism (*MAPRE1*), and cardiac remodeling (*PLZF*). Furthermore, a linkage disequilibrium analysis revealed correlations with other cardiometabolic traits, such as childhood obesity, type 2 diabetes, coronary artery disease, and levels of fasting insulin, HDL cholesterol, and total triglycerides [18].

Smaller gene association studies have also found associations between PCOS diagnosis and genes involved in metabolic risk. An analysis of a Chinese cohort of 744 cases and 895 controls identified three loci associated with several genes of interest [19], including *THADA*, which is currently under investigation as a potential therapeutic target for its role in insulin resistance, thermogenesis, and pancreatic β-cell function [20]. A subsequent European GWA study using a discovery set of 984 cases and 2964 controls reported three loci of significance associated with gonadotropin signaling genes, specifically *FSHB*, and reproduced the association with *THADA* [21]. Interestingly, a secondary analysis of this cohort implemented the unsupervised phenotypic clustering of subjects based on anthropometric, reproductive, and metabolic traits, and it found that in the subset of subjects with a metabolic predominant phenotype, an additional significant locus was identified involving *KCNH7*/*FIGN* and topologically interacting with *GRB14*, a gene implicated in insulin receptor signaling and type 2 diabetes [22]. Another study found that variants of *PON1* (encoding paraoxonase-1, a hydrolase involved in low-density lipoprotein (LDL) metabolism), *IGF2* (encoding insulin-like growth factor 2) had higher rates of homozygosity in a cohort of 72 patients with PCOS compared to 42 controls [23]. Both these genes have been previously associated with insulin resistance and obesity.

Epigenetic mechanisms of PCOS pathogenesis are a growing area of research, for which several studies of human tissues have been performed. One predominant hypothesis attributes the PCOS phenotype to global genomic hypomethylation. Pan et al. found that between 110 cases and 119 controls, 5-methylcytosine profiling of granulosa cell methylomes found a 25% reduction in global methylation, and differential hypomethylation found in promoter regions associated with lipid and steroid synthesis [24]. Another study using a mouse model of PCOS not only found the hypomethylation of genes implicated in other differential DNA methylation studies of humans, but that treatment with a methyl-group donor resulted in the correction of transcriptional, hormonal, and metabolic derangements associated with the PCOS phenotype [25]. One small epigenomic study comparing CpG methylation patterns of adipose tissue from 64 cases and 30 controls identified differentially methylated CpG sites associated with genes, which have been previously associated with type 2 diabetes and obesity via GWA studies (*ARF5*, *GALNTL4*, *R3HDML*, *PSMD6*, and *PROX1*) [26]. In the offspring of affected women, differential methylation patterns have been identified in regulatory regions associated with metabolic genes, notably genes associated with leptin (*LEPR*, *LEP*) and adiponectin (*ADIPOR2*) signaling pathways [27].

Pre-clinical animal models of mice, zebrafish, and sheep have been critical in generating burgeoning hypotheses on epigenetic mechanisms in PCOS pathogenesis and heritability, including heritable modifications across multiple generations and the role of the in-utero environment on epigenetic programming [25,28,29,30,31]. These are areas of active investigation. Table 1 summarizes the possible implicated genes and their role in the association between PCOS and CVD.

### 4.2. The Role of Androgens and Insulin, and Their Interplay

The molecular intersection between PCOS and cardiometabolic disorders, largely mediated by sex hormones, primarily involves mechanisms through which androgens contribute to CVD. Testosterone and androstenedione bind to androgen receptors present in various tissues, including muscle, liver, adipose tissue, and the cardiovascular system, leading to subclinical cardiovascular pathologies, such as increased arterial stiffness, carotid intima-media thickness, and coronary artery calcification [32]. To explore the androgen-driven cardiovascular risk in women with PCOS, a study compared the incidence of multiple cardiometabolic disorders in women with varying androgenic profiles [2]. Women who remained hyperandrogenic throughout the follow-up period, as well as women who transitioned from a hyper- to a normoandrogenic status, exhibited a significantly higher prevalence of hypertension and lower HDL cholesterol levels despite adjusting for BMI compared to consistently normoandrogenic women. These differential risks, however, were not noted for subclinical outcomes, with calcium scores and prevalence of coronary plaques being the same across all groups [2].

At the molecular level, androgens contribute to the predisposition to cardiometabolic disorders through several pathways. Regarding obesity, the role of androgens is gender-selective [9,33]. While androgens suppress adipogenesis in males, they may promote visceral preadipocyte proliferation in females, although the precise mechanism remains unclear [34]. Proposed pathways include the APOBEC3b (apolipoprotein B mRNA-editing enzyme catalytic subunit 3B), CCNA2 (cyclin A2), and PRC1 (protein regulator of cytokinesis 1) pathways [34]. Furthermore, androgens may induce lipogenesis by inhibiting catecholamine-mediated lipolysis, thereby contributing to visceral adiposity [35]. This effect is further amplified by the presence of androgen-synthesizing enzymes in adipose tissue, leading to local androgen production and self-perpetuating adipogenesis, a phenomenon particularly evident in PCOS [36].

Testosterone, as a primary androgen, has also been implicated in the promotion of hypertension in women, with several mechanistic pathways described, including modulation of matrix metalloproteinase-9, 20-hydroxyeicosatetraenoic acid, and vascular endothelial growth factor (VEGF) [37,38]. Additionally, androgens can activate the renin–angiotensin–aldosterone system, a key regulator of blood pressure [39]. Androgens can also contribute to chronic low-grade inflammation, oxidative stress, and endothelial dysfunction, impairing vascular dilation and leading to elevated blood pressure [40]. Furthermore, androgens may exert epigenetic effects by binding to androgen receptors on the promoter regions of specific microRNA (miRNA) genes, activating their transcription and increasing miRNA expression. In women with PCOS, elevated androgen levels are associated with increased expression of miR-155, miR-223, and miR-374, which are implicated in insulin resistance, inflammation, and metabolic disturbances [41]. Moreover, low levels of sex hormone-binding globulin (SHBG), commonly observed in PCOS, correlate with higher coronary artery calcium scores and increased cardiovascular risk, particularly in postmenopausal women [42,43]. Of note, circulating SHBG levels are inversely associated with obesity, insulin resistance, metabolic syndrome, type 2 diabetes, and gestational diabetes [44,45,46].

In relation to insulin resistance, several molecular mechanisms contribute to the impaired insulin signaling observed in both PCOS and cardiometabolic disorders. These include defects in insulin receptor tyrosine phosphorylation, which reduces downstream signaling and promotes insulin resistance [47]. In PCOS, defects in insulin signaling involve decreased insulin-dependent receptor tyrosine phosphorylation and increased constitutive serine phosphorylation of the receptor, ultimately resulting in impaired cellular glucose uptake [5,48]. On a genetic level, the *INSR* and *FBN3* genes have been identified as key contributors to insulin resistance in PCOS, encoding proteins involved in insulin receptor function and extracellular matrix formation, respectively [49,50]. Additionally, the presence of visceral adiposity in women with PCOS phenotype A exacerbates insulin resistance, further enhanced by excess androgen production [51]. Women with obesity and PCOS, in particular, exhibit hepatic insulin resistance, which impairs glycogen synthesis while promoting gluconeogenesis, resulting in hyperglycemia [52]. Intriguingly, insulin signaling in the polycystic ovary, and potentially in other tissues, is selective. Cultured luteinized granulosa cells from women with PCOS exhibit hyperresponsiveness to insulin’s mitogenic actions, promoting cell growth and androgen production, but show resistance to insulin’s metabolic effects such as glucose incorporation into glycogen [53]. In support of this, the insulin sensitizer troglitazone has been shown to reverse both phenomena in the same study [53].

**Figure 2:**
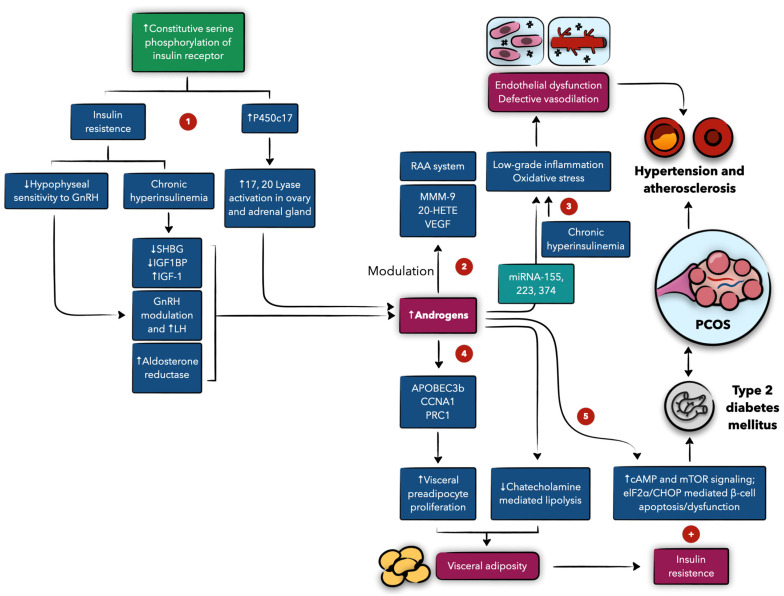
The molecular interaction between insulin and androgens in the pathophysiology of polycystic ovary syndrome (PCOS) and cardiometabolic disease. 1. Defects in insulin receptor and signaling contribute to the insulin resistance seen in PCOS as well as increased androgen production. 2. Androgens modulate several pathways involved in vascular remodeling, potentially contributing to hypertension. 3. Androgens, through miRNA upregulation, and hyperinsulinemia can contribute to states of inflammation and oxidative stress, eventually leading to endothelial dysfunction, and potentially hypertension and atherosclerosis. 4. Androgen are involved in several cascades that promote visceral adiposity through inducing lipogenesis and inhibiting lipolysis. 5. Besides their contribution to insulin resistance, androgens are involved in signaling pathways that predispose to type 2 diabetes mellitus.

The synergistic interaction between androgens and insulin signaling further amplifies the risk of cardiovascular sequelae in PCOS. Hyperinsulinemia is known to stimulate ovarian androgen production in patients with PCOS, and these women may be genetically predisposed to both phenomena, leading to interconnected molecular cascades. For instance, serine phosphorylation of the insulin receptor may induce hyperandrogenemia by upregulating P450c17 activity, the key enzyme in androgen biosynthesis, thereby increasing ovarian and adrenal androgen production [54,55,56]. Additionally, insulin influences the hypothalamic–pituitary axis, increasing gonadotropin-releasing hormone expression and release, which in turn boosts luteinizing hormone (LH) secretion and androgen synthesis [57]. Insulin resistance at the pituitary level further disrupts gonadotropin secretion and ovarian function [58,59]. Figure 2 summarizes the molecular interaction between insulin and androgens in the pathophysiology of polycystic ovary syndrome (PCOS) and cardiometabolic disease.

Moreover, hyperinsulinemia suppresses SHBG production, leading to increased free testosterone levels and elevated insulin-like growth factor 1 levels, which promote ovarian growth and androgen synthesis [60,61]. Insulin also enhances basal and adrenocorticotropic hormone-mediated expression of P450c17 in adrenal cells, contributing to increased androgen production. Peripherally, insulin upregulates type 1C3 aldosterone reductase in adipocytes, converting androstenedione to testosterone and further contributing to the androgen excess seen in PCOS [47,62].

Androgens themselves are also implicated in the predisposition to diabetes and insulin resistance. Testosterone exposure in preadipocytes from healthy women alters insulin signaling by affecting protein kinase C phosphorylation, leading to reduced expression of insulin-induced GLUT-4 and glucose uptake while sparing mitogenic pathways [47,51]. These findings support the hypothesis that insulin dysfunction in PCOS may primarily affect metabolic rather than mitogenic pathways. Elevated androgen levels are associated with higher glycosylated hemoglobin and serum insulin levels, while insulin sensitivity decreases with an increased free androgen index [63]. Gender-specific differences in the androgen effects on glucose metabolism have also been observed [64,65], with hyperandrogenemia promoting hyperinsulinemia through androgen-dependent pathways and the activation of cAMP and mTOR signaling in female mice [66]. Furthermore, testosterone can induce β-cell apoptosis and dysfunction through pathways involving elF2α/CHOP, endoplasmic reticulum stress, and mitochondrial dysfunction [67]. An additional mechanism underlying elevated androgen levels and insulin resistance in PCOS involves antimüllerian hormone (AMH), which is frequently elevated in PCOS [68]. Previous in vitro studies have indicated that AMH may inhibit aromatization in human granulosa cells, leading to the increased synthesis of androgens within the follicle [69]. This rise in androgen production subsequently contributes to the development of insulin resistance, as previously discussed. These findings, though mostly originating in experimental animal models and in vitro human studies, give valuable insights to the possible mechanistic aspects grounding the strong association between PCOS and diabetes.

In the broader context of CVD, hyperandrogenism in PCOS has been implicated in left ventricular hypertrophy and potential cardiac dysfunction, as demonstrated in experimental models with prenatal androgen exposure [70]. This may occur through direct interactions with calcium-calmodulin-dependent protein kinase II and myocyte-enhancer factor 2 or indirectly through the activation of cardiac insulin signaling and the upregulation of the phosphoinositide 3-kinase/AKT/mTOR pathway [71,72]. Although these mechanisms are biologically plausible, their confirmation in human tissues remains pending. Additionally, chronic hyperinsulinemia can activate inflammatory pathways, increase proinflammatory cytokines and reactive oxygen species (ROS) levels, and impair endothelial function, reducing nitric oxide production and contributing to vascular dysfunction [73,74].

### 4.3. Adipokines

Adipokines are cell-signaling molecules primarily secreted by adipose tissue. While they are well known for their involvement in obesity-related inflammation, recent studies have highlighted their role in the pathophysiology of PCOS (Table 2), further linking PCOS to cardiometabolic disorders [75,76,77]. Although adipokines are predominantly produced by adipocytes, the dysregulation of these molecules has also been observed in individuals without obesity but with PCOS [77].

Substantial evidence supports the involvement of adipokines in the metabolic disturbances associated with PCOS, particularly in relation to insulin resistance [19,75,78]. For example, chemerin, a proinflammatory chemoattractant, is present at elevated levels in women with PCOS and is associated with insulin resistance [79]. Chemerin seems to participate in a feed-forward loop, promoting both systemic and local insulin secretion in the human uterus and stromal cells [76]. Leptin, another adipokine, is thought to contribute to the pathogenesis of PCOS by stimulating insulin secretion from adipose tissue, thereby exacerbating insulin resistance [80,81]. Conversely, omentin, a cardioprotective adipokine, is found in reduced concentrations in conditions like type 2 diabetes mellitus and PCOS, both of which are characterized by insulin resistance [82]. Adiponectin, which normally enhances tissue sensitivity to insulin and promotes lipid oxidation via AMPK signaling, is significantly diminished in patients with PCOS [75].

Certain adipokines may also play a role in the hyperandrogenism observed in approximately 80% of women with PCOS. In vitro studies suggest that elevated levels of resistin in PCOS may upregulate the activity of 17α-hydroxylase, contributing to excessive androgen production [76,79]. Leptin, under physiological conditions, enhances aromatase expression in granulosa cells, but this effect is impaired in PCOS, leading to increased circulating androgen levels [83]. This has led to the hypothesis of leptin resistance in PCOS, potentially mediated by the RNA-binding protein Sam68 [76,83]. Table 2 summarizes alterations in adipokine levels and their associated metabolic effects in the context of PCOS.

The inflammatory component of PCOS is further supported by the role of adipokines in the syndrome’s pathophysiology. Elevated resistin levels in PCOS induce macrophage-mediated production of proinflammatory cytokines, such as tumor necrosis factor (TNF)-α, interleukin (IL)-6, and IL-12 [76]. Additionally, omentin, which has protective effects on cardiovascular health, is downregulated in the presence of high testosterone and inflammatory cytokines like TNF-α and IL-6 in patients with PCOS, suggesting that inflammatory milieus seen in PCOS may adversely affect cardiovascular function [76]. However, whether this subclinical inflammatory state is a significant contributor to CVD in PCOS requires further investigation.

Adipokine levels in PCOS have been shown to be modulated by both behavioral and pharmacologic interventions typically aimed at improving cardiovascular health. For instance, high-intensity interval training has been shown to increase adiponectin levels in patients with PCOS [84]. Similarly, metformin treatment has been reported to reduce the levels of vaspin and chemerin after six months of therapy [76,85], as well as lower elevated levels of AMH in women with PCOS [86]. Additionally, circulating omentin concentrations increase following treatment with metformin or combined oral contraceptives (COCs) in patients with PCOS [76]. These findings suggest that adipokines could serve as potential biomarkers for assessing treatment efficacy in PCOS.

### 4.4. PCOS and Atherosclerosis

Atherosclerosis, an inflammatory process affecting large arteries, leads to the formation of subendothelial plaques composed of fibrous tissue, calcium deposits, and lipids [87]. The heightened risks of obesity, dyslipidemia, insulin resistance, and hypertension associated with PCOS contribute to endothelial damage and the development of atherosclerotic plaques, thereby increasing CVD risk [88]. Hyperandrogenism in PCOS, particularly elevated testosterone, is linked to endothelial dysfunction and increased risk of atherosclerosis [80,88]. Elevated endothelin-1, a biomarker of endothelial injury, has been observed in women with hyperandrogenic PCOS phenotypes (A–C), independently of BMI [89]. SHBG, which exerts cardioprotective effects by mitigating oxidative stress and vascular damage, is typically reduced in individuals with PCOS [80,90]. This imbalance in sex hormone regulation in PCOS contributes to an altered cardiovascular risk profile.

Women with PCOS have a twofold increased risk of coronary artery calcification compared to women without PCOS [11]. A prospective cross-sectional study found that PCOS was the strongest predictor of elevated carotid intima-media thickness after adjusting for age, smoking status, and BMI [91]. Endothelial dysfunction in PCOS is also demonstrated by reduced flow-mediated dilation of the brachial arteries compared to age-matched controls [92]. A meta-analysis encompassing over 5000 participants showed that women with PCOS, aged 14–56 years, exhibited significantly greater structural and functional markers of atherosclerosis, including thicker carotid intima-media thickness, lower flow-mediated dilation, lower nitroglycerin-mediated vasodilation, and increased coronary artery calcification [93]. These findings suggest that PCOS may likely contribute to atherosclerosis independently of obesity and other cardiometabolic comorbidities.

Subclinical atherosclerosis develops at a significantly earlier age in women with PCOS compared to the general population. Atherosclerotic plaques have been detected in the carotid intima of patients with PCOS as early as adolescence [94]. Moreover, inflammatory markers associated with atherosclerosis, such as C-reactive protein (CRP), IL-6, TNF-α, leptin, and adiponectin, are elevated at younger ages in women with PCOS, particularly in those who are obese [89,95]. Both pre- and postmenopausal women with PCOS have higher coronary artery calcium scores than their peers after adjusting for age and BMI [88]. Interestingly, the presence of atherosclerosis in patients with PCOS does not decrease after menopause [96]. Furthermore, cardiometabolic biomarkers, including insulin resistance, hypertension, and BMI, often worsen during and after menopause in women with PCOS [96]. While the increased prevalence of atherosclerosis and cardiovascular risk factors in PCOS may raise concerns about cardiovascular mortality in these patients, existing data on the actual impact on lifespan are inconclusive [97,98]. Additional research is needed to clarify the long-term cardiovascular implications of PCOS on women’s health.

### 4.5. Angiogenesis

Angiogenesis is a critical process in both PCOS and CVD, playing a significant role in the pathogenesis and progression of each condition [99]. In the ovaries, angiogenesis and vascular regression typically occur during each menstrual cycle; however, this process is disrupted in PCOS [100]. Notably, increased vascularization of the ovarian stroma is observed in individuals with PCOS [101,102]. This enhanced vascularization alters oxygen and nutrient delivery, which may contribute to the formation of ovarian cysts in PCOS. One of the most well-studied angiogenic factors in this context is VEGF. In PCOS, there is a positive correlation between VEGF levels and ovarian blood flow [101], and *VEGF* gene polymorphisms have been identified in various PCOS populations [103,104,105]. Other angiogenic factors, including basic fibroblast growth factor [106], transforming growth factor-β [107], and placental growth factor [108], are also implicated in the condition.

Angiogenesis is similarly pivotal in CVD pathogenesis. While myocardial infarction and atherosclerosis are generally associated with impaired angiogenesis, characterized by reduced blood flow and oxygen delivery, subsequent remodeling and the upregulation of pro-angiogenic factors attempt to compensate for hypoxia and ischemia. These factors may also increase the exposure of atherosclerotic plaques to inflammatory cells, potentially exacerbating plaque development [109]. VEGF is a key player in this process. Additionally, both basic fibroblast growth factor and transforming growth factor-β are implicated in CVD, with basic fibroblast growth factor serving as a predictive biomarker for future CVD events in individuals with type 2 diabetes [110,111]. Ultimately, both PCOS and CVD are characterized by altered angiogenesis. Given the upregulation of angiogenic factors in both conditions, it is speculated that the angiogenesis observed in one condition could exacerbate or synergistically contribute to angiogenesis in the other, although this potential interaction remains to be explored.

### 4.6. The Reproductive Microbiome

An increasing body of evidence suggests that the chronic, low-grade inflammatory state observed in PCOS, which may contribute to insulin resistance and CVD, could potentially have its origin, at least in part, within the local vaginal microbiome [112]. *Bacteroides* species are found in high abundance within the microbiome of patients with PCOS, where they modulate bile acid metabolism by reducing the secretion of glycodeoxycholic acid and tauroursodeoxycholic acid. This alteration in bile acid secretion subsequently influences the release of proinflammatory cytokines such as IL-22 [113]. IL-22, a cytokine involved in host defense regulation and tissue regeneration, is notably reduced in individuals with PCOS [114,115]. Furthermore, elevated levels of both *Prevotella* and *Bacteroides* species have been directly associated with insulin resistance [116]. In animal studies, Qi et al. demonstrated that oral administration of *Bacteroides* bacteria in mice induced insulin resistance, disrupted the estrous cycle, and altered ovarian morphology [115]. These findings suggest a potential role for these bacterial species in the pathogenesis of PCOS and its associated metabolic sequelae.

The presence of healthy vaginal microbiota is essential for preventing pathological inflammatory responses, and a deeper understanding of its composition may provide novel therapeutic insights for PCOS. A review by Rodriguez Paris et al. highlighted a study in which fecal microbiota transplantation with *Lactobacillus* species in patients with PCOS restored follicular morphology and reduced levels of testosterone and androstenedione [117]. In another study, microbial translocation from healthy individuals to obese subjects led to a reduced BMI and improved insulin sensitivity, although this has not been specifically demonstrated in patients with PCOS [112]. Furthermore, Zhao et al. explored the potential use of traditional Chinese medicine, specifically *Ophiopogon japonicus* (dwarf lilyturf or mondograss), in the treatment of PCOS. This plant was shown to decrease *Bacteroides* levels within the vaginal microbiome [118]. While fecal microbiota transplantation shows promise as a potential treatment for PCOS, additional clinical trials are necessary to fully understand its therapeutic benefits in human patients [114].

### 4.7. The Role of Vitamin D, Oxidative Stress, and Environmental Factors

Social determinants, such as food insecurity, lower socioeconomic status, sedentary behavior, and obesity, are recognized as factors that increase the risk of PCOS [119,120,121]. However, lifestyle modifications targeting diet and physical activity have been shown to influence PCOS pathophysiology at the molecular level [122]. Diets with a high glycemic index have been linked to the onset of PCOS. Hyperglycemia itself contributes to the generation of ROS by stimulating proinflammatory pathways, such as the activation of TNF-α and NF-kappa B (NF-ĸB). This inflammatory response exacerbates insulin resistance, creating a detrimental feedback loop that perpetuates hyperglycemia-induced insulin resistance [123].

Vitamin D deficiency has been identified as a significant factor in the development and exacerbation of PCOS, with lower vitamin D levels correlating with the severity of its signs and symptoms and repletion of vitamin D to normal levels demonstrating an improvement its signs and symptoms [124,125,126]. Similarly to insulin resistance, vitamin D deficiency is associated with decreased levels of SHBG, leading to elevated free testosterone. One study demonstrated that vitamin D supplementation resulted in a reduction in testosterone and androstenedione levels in patients with PCOS after 3 months of treatment [125]. Furthermore, low vitamin D status is linked to insulin resistance and increased BMI, potentially due to its role in reducing insulin secretion and receptor activity [125,127]. A study by Chakraborty et al. evaluating the association between vitamin D profiles and different indices and clinical features of PCOS, including AMH levels, insulin resistance, hyperandrogenism, and obesity indices found a direct correlation between vitamin D and AMH levels in PCOS phenotype A. In addition, an agonistic relationship between vitamin D and insulin resistance was present in the study population [128].

## 5. Clinical Implications

### 5.1. Screening and Diagnostic Implications

#### 5.1.1. Anthropometric Markers

PCOS is associated with significant cardiometabolic comorbidities, highlighting the importance of timely identification and management of CVD risk in affected individuals. Anthropometric markers provide non-invasive, cost-effective, and reliable indicators of cardiometabolic risk in patients with PCOS. Waist circumference, for example, is often elevated in individuals with PCOS, even in the absence of obesity, and serves as a marker for abdominal adiposity—a known risk factor for CVD [129]. According to Wild et al., a waist circumference ≥88 cm in non-Asian women and ≥80 cm in East/South Asian women is considered elevated and should be interpreted as an indicator of increased CVD risk in the management of PCOS [129]. Further studies have demonstrated that individuals in the highest CVD risk category typically exhibit increased waist circumference, waist-to-hip ratio, and upper arm circumference [130]. These measurements also show a positive correlation with obesity [130]. Additionally, while BMI remains an essential parameter for evaluating cardiovascular risk in patients with PCOS, CVD biomarkers are elevated in PCOS regardless of BMI, but their concentrations are further amplified in the presence of obesity [131].

#### 5.1.2. Lifestyle and Behavioral Screening

While PCOS increases patients’ risk of adverse CVD outcomes in its own right, guidelines also support assessment of other lifestyle factors that might compound CVD risk in patients with PCOS. For example, according to consensus at the European Society of Human Reproduction and Embryology, psychosocial stressors, barriers to exercise, and smoking status may contribute to a patient’s cardiovascular outcomes and may require additional, personalized counseling [131]. Screening for obstructive sleep apnea has been recommended to mitigate pulmonary hypertension or right-heart damage secondary to PCOS-related comorbidities [132]. Anxiety and depression are prevalent to greater extents in people with PCOS than the general population, and these conditions are significantly associated with CVD risk. As such, behavioral health assessments play an important role in screening for CVD risk factors among patients with PCOS [131].

#### 5.1.3. Metabolic Screening

Given the comorbid conditions commonly associated with PCOS, it is recommended that newly diagnosed patients undergo evaluation for glucose tolerance and dyslipidemia. This can be achieved through glucose tolerance testing or measurement of hemoglobin A1c, and lipid panels, respectively [133]. Non-HDL cholesterol, in particular, is a valuable indicator of long-term cardiometabolic risk [131]. Additionally, the index of central obesity and non-alcoholic fatty liver disease indices have been significantly correlated with CVD risk in patients with PCOS [130]. Consequently, risk stratification in PCOS may benefit from the inclusion of both anthropometric and hepatic measurements [130]. Routine blood pressure assessment is also essential for detecting hypertension in patients with PCOS [130].

#### 5.1.4. Atherosclerotic Cardiovascular Disease Risk Stratification

Subclinical vascular disease is a predictor of adverse cardiovascular events, and individuals with PCOS are at an increased risk of subclinical vascular disease from an early age [94]. Notably, the use of atherosclerotic CVD risk calculators can help contextualize lipid levels, blood pressure, and other factors when assessing long-term cardiovascular risk. For individuals with indeterminate or intermediate risk, coronary artery calcification scoring via CT imaging may be performed [134]. Statin therapy is recommended for those with a coronary artery calcification score above 100 or greater than the 75th percentile for age, sex, and race [88]. Carotid intima-media thickness is another measure used to assess atherosclerotic CVD risk, though it is considered less reliable than coronary artery calcification scoring [88]. While evidence of subclinical atherosclerosis may emerge early in patients with PCOS, routine screening is not advised before the age of 40 due to the lack of clear clinical benefit [88].

#### 5.1.5. Androgens as Biomarkers

Hyperandrogenism in PCOS is linked to increased cardiovascular risk and metabolic dysfunction. Several studies have highlighted the utility of androgens and SHBG as biomarkers for cardiometabolic risk. For instance, the Multi-Ethnic Study of Atherosclerosis (MESA), which included over 2750 postmenopausal women, found that an elevated testosterone-to-estrogen ratio and reduced SHBG levels were associated with a higher progression of coronary artery calcification [43]. Similarly, the Study of Women’s Health Across the Nation (SWAN), which included 3297 pre- and perimenopausal women, showed that low SHBG levels and a high free androgen index value were positively correlated with insulin resistance, inflammation, and dyslipidemia [132]. Androstenedione also exhibits a strong negative correlation with insulin sensitivity [135]. Elevated levels of both testosterone and androstenedione have been linked to the highest cardiometabolic risk in patients with PCOS [132,135]. Additionally, a higher testosterone-to-dihydrotestosterone ratio in PCOS individuals correlates with insulin resistance, dyslipidemia, and adverse liver parameters [136]. O’Reilly et al.’s study further indicated that patients with normal testosterone levels, but elevated androstenedione, are at an increased risk for metabolic syndrome [135].

#### 5.1.6. Genetics

Although genetic screening is not yet routine practice, genetic analysis may become an important diagnostic tool in managing PCOS in the future. Several gene classes have been implicated in the cardiometabolic risk profile of patients with PCOS. For instance, the calpain 10 gene appears to contribute to metabolic syndrome in PCOS by promoting insulin resistance [132]. Additionally, polymorphisms in the platelet-activating factor acetylhydrolase gene have been linked to variations in plasma lipoprotein levels and insulin insensitivity in patients with PCOS [132]. Recent large-scale GWA studies have identified common loci responsible for the clinical features observed across various PCOS phenotypes [18,88]. These loci have been used to calculate polygenic risk scores (PRSs) [137]. In a study by Zhu et al., PRSs were retroactively calculated for the male family members of patients with PCOS and were found to correlate with cardiometabolic risk factors including obesity, hypertriglyceridemia, and type 2 diabetes [137]. This suggests that the PRS may serve as a potential marker for identifying cardiometabolic risk in individuals with PCOS, independently of ovarian dysfunction. Furthermore, the heritable nature of PCOS underscores the importance of proactive screening and management for first-degree relatives of individuals with PCOS. According to a study by Yilmaz et al., first-degree relatives of individuals with PCOS are at a higher risk for cardiometabolic diseases compared to the general population [138]. Therefore, the presence of PCOS in a patient’s family history should be considered when stratifying the risk for metabolic syndrome.

#### 5.1.7. Emerging Biomarkers

AMH, as previously stated, has been implicated in the pathogenesis of PCOS, and its association with cardiometabolic comorbidities remains under investigation [139,140]. Feldman and colleagues reported that lower AMH levels in young individuals with PCOS are correlated with the presence of metabolic syndrome [141], a finding that has been supported by recent systematic reviews [142]. Since AMH levels are typically elevated in patients with PCOS, this may indicate a specific PCOS phenotype that could be more prone to cardiometabolic comorbidities. While the exact relationship between AMH and the cardiovascular system remains unclear, animal studies have shown that AMH is present in regions outside the reproductive system, including the lungs and brain, hinting at a potential role in processes beyond reproductive physiology [143,144]. Given PCOS’s inflammatory nature, chemokines are being explored as potential biomarkers for risk stratification. CRP, a known CVD marker, has been found to be elevated in individuals with PCOS [132]. Moreover, patients with PCOS often exhibit increased levels of inflammatory molecules such as TNF-α, IL-6, IL-8, and IL-18 [145], with IL-18 being particularly strongly associated with adverse cardiovascular outcomes [146]. However, these chemokines reflect systemic inflammation, and further research is required to establish their specificity in assessing cardiometabolic risk in PCOS. Adipokines, another class of cell-signaling molecules, may also contribute to PCOS cardiometabolic risk assessment. For example, omentin, due to its role in the atherosclerotic pathway, has been proposed as a potential biomarker of CVD risk [147]. Additionally, adiponectin, which is well established as a biomarker for type 2 diabetes and coronary artery disease, may be useful in future studies examining cardiometabolic risk in PCOS [75,148].

## 6. Preventative and Therapeutic Implications

### 6.1. Behavioral and Lifestyle Modifications

First-line treatment approaches for PCOS typically involve lifestyle and behavioral modifications, which primarily target weight loss and insulin resistance, as these are key contributors to the pathophysiology of PCOS. Notably, 69% of individuals with PCOS are obese, and 64% meet the criteria for metabolic syndrome [149,150]. There is evidence suggesting that a weight loss of 5–10% in patients with PCOS is associated with improvements in clinical and biochemical hyperandrogenism as well as ovulatory dysfunction [151].

Very low calorie diets, including the ketogenic diet, characterized by a daily carbohydrate intake of less than 50 g, along with varying amounts of fat and protein based on ideal body weight, have been shown to significantly improve weight loss outcomes in individuals with PCOS [152]. The ketogenic diet also positively affects reproductive hormone profiles, including a reduced LH/follicle stimulating hormone (FSH) ratio (d −0.851; 95% CI −1.015, −0.686; *p* < 0.001), decreased serum free testosterone (d −0.223; 95% CI −0.328, −0.119; *p* < 0.001), and increased SHBG levels (d 9.086; 95% CI 3.379, 14.792; *p* = 0.002) [153,154]. The observed improvements in weight loss and reproductive hormones are thought to stem from increased metabolic efficiency, which is facilitated by enhanced lipolysis, increased protein intake that promotes satiety, and elevated ketone production that suppresses appetite. Moreover, improved insulin sensitivity is believed to be the mechanism through which the LH/FSH ratio is decreased, as hypothalamic–pituitary–ovarian/adrenal axis dysfunction is often exacerbated in hyperinsulinemic states.

High serum insulin levels and large glucose loads are known to be proinflammatory, promoting the production of ROS [155,156]. Emerging evidence also suggests that reactive hypoglycemia, often observed in PCOS and other metabolic disorders, contributes to hypothalamic–pituitary–ovarian/adrenal axis dysfunction via adrenal hormone secretion, particularly cortisol and dehydroepiandrosterone (DHEA). This dysfunction may be mitigated by reducing simple carbohydrate intake in a calorie-restricted diet and replacing it with whey protein, which suppresses ghrelin for longer periods compared to simple carbohydrates. This dietary change helps minimize the risk of reactive hypoglycemia and its associated downstream effects [150,157].

In addition, behaviors such as smoking, alcohol use, and recreational drug use have been linked to elevated levels of proinflammatory markers, decreased antioxidant levels (e.g., β-carotene), and poor PCOS outcomes, including reduced fertility and increased hirsutism [158]. Research shows that individuals with PCOS are more likely to engage in these behaviors compared to their peers, highlighting the importance of targeted counseling and interventions aimed at reducing inflammation and improving PCOS outcomes [159].

### 6.2. Therapeutic Implications

#### 6.2.1. Pharmacological Antioxidants

Given the significance of inflammation, increased oxidative stress and decreased antioxidants underlying the PCOS disease process, studies have investigated the use of pharmacologic antioxidants in patients with PCOS [160]. As with diet and lifestyle modifications, pharmacologic antioxidants have been focused on improving outcomes by way of insulin and weight regulation. The more popular antioxidants include coenzyme Q10 (CoQ10), vitamin E, vitamin D, and inositols. A 2022 study showed that 100 mg daily of coenzyme q10 supplementation for 12 weeks resulted in decreased CRP, total testosterone, DHEA-sulfate, hirsutism, total antioxidant capacity, and Malondialdehyde levels [161]. CoQ10 stabilizes cell membranes and prevents mitochondrial dysfunction, thus averting oxidative stress [162].

A 2022 metanalysis found that Vitamin E supplementation reduced fasting glucose, fasting insulin, homeostasis model assessment of insulin resistance (HOMA-IR), total cholesterol, LDL-cholesterol, triglycerides, total testosterone, and increased SHBG [163]. Studies have suggested vitamin E improves insulin sensitivity by upregulating an endogenous ligand involved in activating peroxisome proliferator-activated receptor gamma (PPAR-γ), which has a role in upregulating adiponectin [164].

Recent studies have found a positive correlation between vitamin D deficiency, dysglycemia, obesity, and PCOS, and a negative correlation between BMI and vitamin D level in patients with PCOS [165,166,167,168]. In a 2022 metanalysis, vitamin D supplementation for 12 weeks in patients with PCOS resulted in an improvement in the levels of total testosterone, CRP, total antioxidant capacity, and Malondialdehyde levels [169]. Vitamin D is thought to attenuate the inflammatory environment in PCOS through its action of inhibiting NF-ĸB, an important transcription factor regulating the inflammatory response to produce TNF-α. Specifically, vitamin D inhibits NF-ĸB, thereby reducing the production of free radicals and proinflammatory cytokines [170].

Inositols (myo-inositol and D-chiro inositol), which are found in the cell membrane, and are involved in cell signaling pathways to transport GLUT4 to the plasma membrane for glucose uptake, have also been studied in PCOS with inconclusive evidence. Because of the role inositol has in regulating maturation and proliferation of granulosa cells, and aromatase synthesis, further research is indicated to clarify the role of inositol supplementation for PCOS treatment [171,172,173].

#### 6.2.2. COCs

COCs are the first-line treatment for patients with PCOS, not desiring fertility, though no formal recommendations have been made regarding the optimal type of COC or duration of treatment. By increasing SHBG levels, serum androgens decrease which in turn improve hirsutism, acne, and menstrual cycle irregularities in patients with PCOS [174,175,176]. Additionally, the estrogen component of COCs decreases LH production, further decreasing free testosterone levels [177]. COCs, however, do not attenuate the adverse cardiometabolic effects of PCOS, and in fact may worsen them as increased serum inflammatory markers, including CRP, LDLs, ICAM-1, TNF-α, and MCP-1 mRNA have been observed with prolonged COC [178,179]. The mechanisms driving these processes have yet to be elucidated.

#### 6.2.3. Metformin

Metformin, an insulin sensitizing agent, is currently recommended as a first line treatment in addition to lifestyle modifications for patients with PCOS who are overweight or have metabolic syndrome [180]. In a systematic review of RCTs, metformin was found to reduce BMI, HOMA-IR, fasting glucose, and menstrual cycle days, compared to a placebo [180]. By increasing insulin sensitivity in the liver and peripheral tissues and reducing gluconeogenesis, metformin decreases hyperglycemia and compensatory β-cell hyperinsulinemia. This ultimately results in decreased circulating androgens by mitigating hypothalamic–pituitary–adrenal axis dysfunction. Additionally, by regulating the hypothalamic–pituitary–adrenal axis, including by regulating hunger and satiety via leptin release, metformin precipitates weight loss [181,182]. Lastly, metformin has also been found to regulate inflammatory hormones including CRP, alleviating the chronic inflammatory processes at play with PCOS [169,183]. On a molecular level, metformin works by changing ATP/AMP levels, activating the AMPK pathway to prevent gluconeogenesis, reduce hyperglycemia and triglyceridemia, and activate T regulatory cell proliferation [184,185,186]. In addition, as previously mentioned, metformin was found to lower elevated levels of AMH in women with PCOS, potentially providing protective effects against AMH-mediated cardiometabolic risks seen in this population [86]. Given the underlying differences in the driving pathophysiological pathways and metabolic derangements of different PCOS phenotypes, management strategies should also be tailored such that the therapeutic intervention can precisely target the involved metabolic pathway. Phenotype D, for example, lacks the classic hyperandrogenism and metabolic derangements seen in other phenotypes, questioning whether standard therapeutic approaches would be as beneficial in women with this phenotype [187]. In this context, whereas the use of metformin is substantial in targeting the clinical phenomena of insulin resistance in patients with phenotypes A, B, and C, these effects would theoretically be less prominent in phenotype D. In fact, no clinical trials have yet compared the effectiveness of metformin between different phenotypes [187].

#### 6.2.4. Statins

Lipid-lowering drugs, namely statins, have been suggested to have potential therapeutic utility in patients with PCOS, either alone or in combination with other therapeutic drugs [188]. For the purpose of this review, statin therapy alone will be discussed. In a review of patients with PCOS receiving atorvastatin, HOMA-IR was significantly reduced [189]. Studies have also found that simvastatin and atorvastatin can reduce androgen levels in patients with PCOS [190]. Statins decrease insulin resistance by selectively inhibiting the activity of 3-hydroxy-3-methylglutaryl coenzyme A reductase and downregulating the mevalonate pathway, which is closely associated with insulin resistance [189]. As discussed previously, regimens that improve insulin resistance frequently improve hyperandrogenism as HPA dysfunction decreases. While more research is needed in this field to establish a clearer recommendation for the duration, dosage, and type of statin therapy (individual or combined), emerging evidence is promising [191].

#### 6.2.5. GLP-1 Agonists

Glucagon-like peptide-1 (GLP-1) receptor agonists, which have been prescribed for diabetes for nearly two decades and more recently for weight loss, have been studied in patients with PCOS with no response to lifestyle modifications. A 2023 study showed that after three months of 0.5 mg subcutaneous weekly injections of semaglutide, a GLP-1 receptor agonists, a mean decrease in body weight of 7.6 kg and a mean BMI loss of 3.1 was seen, with few reported side effects. Additionally, 80% of patients had at least a 5% decrease in their body weight, insulin basal values decreased, and HOMA-IR improved which was often associated with the normalization of menstrual cycles [192].

GLP-1 receptor agonists cause heightened satiety, reduced appetite, and appetite regulation by acting on L cells of the small intestine to cause glucose-dependent insulin release and contribute to lowering insulin resistance [193]. GLP-1 receptor agonists have demonstrated efficacy in reducing glycated hemoglobin levels and therefore longstanding hyperglycemia, which as previously stated causes the upregulation of inflammatory cytokines [194]. While the precise mechanism by which GLP1-RAs mitigate inflammation is unknown, it is thought to be related to T regulatory cell stabilization through cAMP/AMPK-dependent signaling [184].

#### 6.2.6. Thiazolidinediones

Thiazolidinediones (TZD) have also been implicated in improving the metabolic profiles in patients with PCOS. TZDs act as agonists of PPAR-γ, thereby increasing the number of peroxisomes in cells, which are involved in the breakdown of toxic substances. Specifically, TZDs promote the uptake of insulin-dependent glucose while decreasing hepatic glucose output [182]. The two primary TZDs currently in clinical use are pioglitazone and rosiglitazone. Rosiglitazone exhibits a 30-fold-higher binding affinity for PPAR-γ but has been associated with an increased risk of cardiovascular events, particularly in patient populations predisposed to CVD [195]. Meta-analyses assessing the effects of TZDs on the metabolic profile of patients with PCOS have yielded positive findings. For instance, TZDs have been shown to decrease HOMA-IR, fasting plasma glucose, triglycerides, and LDL, while simultaneously increasing HDL cholesterol [196]. In terms of sex hormones, TZDs have been associated with reduced 17-hydroxyprogesterone secretion in response to leuprolide administration [197], suggesting that TZDs may mitigate the hyperandrogenism typically observed in PCOS.

The comparative efficacy of TZDs relative to metformin, as well as their potential as adjuncts to metformin therapy, has also been an area of interest. Head-to-head studies indicate that TZDs and metformin have comparable effects on fasting glucose and HOMA-IR [182]. However, in a separate analysis, when pioglitazone and rosiglitazone were compared to metformin, pioglitazone and metformin appeared to equally lower testosterone levels in patients with PCOS, while rosiglitazone demonstrated less efficacy in this regard but was superior to metformin in decreasing LDL levels. As an adjunct to metformin, TZDs further lower triglycerides levels [196], though metformin monotherapy is more effective in reducing weight and BMI in patients with PCOS [182]. Ultimately, while TZDs are less commonly used than other therapies such as metformin, they offer significant benefits in improving metabolic profiles in PCOS, both as monotherapy and as adjunctive treatment. Table 3 summarizes the potential therapeutics used to address the metabolic phenomena seen in PCOS.

#### 6.2.7. Surgical Interventions (e.g., Bariatric Surgery)

Bariatric surgeries, including Roux-en-Y gastric bypass, vertical sleeve gastrectomy, and gastric banding have been very popular for weight reduction in obese patients. However, with the arrival of GLP-1 receptor agonists the popularity of bariatric surgery is likely to wane and be used more specifically to those who fail or who lack access to GLP-1 receptor agonists due to lack of affordability. Given the strong association between overweight/obesity and PCOS, bariatric surgeries have been investigated for treatment of patients with PCOS demonstrating promising evidence [198]. In a study of patients with PCOS and obesity with a BMI of >40 who underwent bariatric surgery, oligomenorrhea was improved in 66% of patients, sonographic improvement of PCOS was seen in 74% of patients, and FSH, testosterone, and DHEA levels were significantly improved one year postoperatively [199]. However, in a retrospective analysis of 32 patients with obesity and PCOS, only anthropomorphic measurements including weight and BMI were consistently statistically significantly improved, whereas hormonal outcomes varied [200]. While more research is indicated, before definitive recommendations for bariatric surgery for the treatment of PCOS can be made, theoretical plausibility exists. Weight loss in the setting of bariatric surgery was previously attributed to decreased stomach volume and an obligatory reduction in caloric intake; however, there is evidence pointing to multi-modal mechanisms of action, including central satiety regulation via ghrelin production and gut hormone regulation [201]. Attenuated reward pathways when high-fat meals are consumed have been reported, specifically via the inhibition of local oleoylethanolamide. Additionally, fibroblast growth factor 19 secretion, which signals an energy replete state at the level of the hypothalamus, and increased bile acid production, which leads to increased fatty acid oxidation and stimulation of GLP-1 and peptide YY in the small intestine, both promote anorexigenic gut hormone production in patients after bariatric surgery [202,203]. Additional research is needed to understand the effects of bariatric surgery on hormonal imbalance in patients with PCOS and mechanistic driving forces.

## 7. Conclusions and Future Directions

PCOS has been extensively studied over the years, revealing distinctive aspects of its pathobiology. This review enhances our understanding of the intersection between PCOS and cardiometabolic disease at multiple molecular levels, proposing novel frameworks that could inform future diagnostic, preventative, and therapeutic strategies. While this review examines this association, it also emphasizes the necessity for further mechanistic and clinical research to elucidate the cellular and molecular processes involved in establishing and discovering reliable predictors of CVD for women with PCOS.

## Figures and Tables

**Table 1:** Potential genes implicated in the association between polycystic ovary syndrome (PCOS) and cardiovascular disease (CVD).

Gene	Role
*INSL4*, *INSL6*	Insulin signaling
*MAPRE1*	Adipocyte function, lipid metabolism
*PLZF*	Cardiac remodeling
*THADA*	Insulin secretion
*KCNH7*	Potassium voltage-gated channel
*FIGN*	Microtubule severing
*GRB14*	Insulin receptor signaling
*PON1*	LDL metabolism
*IGF2*	Encodes insulin-like growth factor 2
*LEPR*, *LEP*	Associated with leptin
*ADIPOR2*	Associated with adiponectin

**Table 2:** Alterations in adipokine levels and their associated metabolic effects in the context of polycystic ovary syndrome (PCOS).

Adipokine	Changes in PCOS	Effects in the Context of PCOS
Chemerin	Increased	Increases insulin resistance; increases insulin secretion in the uterus and stromal cells
Leptin	Increased	Stimulates insulin secretion from adipose tissue; in PCOS, impairs aromatase expression in granulosa cells thereby increasing circulating androgen levels
Omentin	Decreased	Has protective effects on cardiovascular health
Adiponectin	Decreased	Enhances tissue sensitivity to insulin; promotes lipid oxidation via AMP-activated protein kinase (AMPK) signaling
Resistin	Increased	Upregulates 17-α-hydroxylase, increasing androgen production; induces macrophage-mediated production of proinflammatory cytokines

**Table 3:** Summary of therapeutics for potential use in PCOS.

Medication	Mechanism of Action	Effects
Antioxidants		
CoQ10	Stabilizes cell membranes, prevents mitochondrial dysfunction and oxidative stress	Decreases total testosterone, dehydroepiandrosterone sulfate (DHEA-S), hirsutism, and sex hormone binding globulin (SHBG) Decreases C-reactive protein (CRP), total oxidant capacity (TAC) and malondialdehyde levels
Vitamin E	Upregulates endogenous ligands involved in activating peroxisome proliferator activated receptor gamma (PPAR-γ)	Decreases total testosterone; increases SHBG Decreases fasting glucose, insulin, and homeostasis model assessment of insulin resistance (HOMA-IR) Decreases total cholesterol, low-density lipoprotein (LDL), triglycerides
Vitamin D	Inhibits nuclear factor kappa B (NF-κB), reducing production of free radical and proinflammatory cytokines	Decreases total testosterone Decreases CRP, TAC, and malondialdehyde levels
Inositols	Involved in cell signaling pathways to transport glucose transporter-4 (GLUT4) to the plasma membrane for glucose uptake, and regulates maturation and proliferation of granulosa cells and aromatase synthesis	Decrease total testosterone Decrease HOMA-IR
Combined oral contraceptives	Increase SHBG and luteinizing hormone (LH) production, reducing serum androgens	Decrease total testosterone
Metformin	Activates the AMPK pathway to prevent gluconeogenesis, improve hyperglycemia, triglyceridemia, and activate T regulatory cell proliferation	Decreases androgen levels Decreases elevated levels of AMH Decreases body mass index (BMI), fasting glucose, increases insulin sensitivity Decreases CRP
Statins	Inhibit 3-hydroxy-3methylglutaryl coenzyme A reductase and thereby decreasing lipid production	Decrease androgen levels Decrease HOMA-IR
Glucagon-like peptide-1 (GLP-1) agonists	Agonist of GLP-1 receptors, heightening satiety, reducing appetite, and regulating appetite by acting on L cells of the small intestine	Decrease insulin basal values and HOMA-IR Reduce glycated hemoglobin levels Accelerate weight loss Decrease inflammatory processes
Thiazolidinediones	Agonist of PPAR-γ, increasing peroxisomes and disposal of insulin dependent glucose, while decreasing hepatic glucose output	Decrease androgen levels Decrease HOMA-IR, fasting plasma glucose Decrease triglycerides, LDL
